# Parental Selection of Preschool Programming in Saudi Arabia: A Qualitative Study

**DOI:** 10.3390/bs12100370

**Published:** 2022-09-29

**Authors:** Mohaned G. Abed, Maha M. Nahshal, Todd K. Shackelford

**Affiliations:** 1 Department of Special Education, Faculty of Educational Graduate Studies, King Abdulaziz University, Jeddah 21589, Saudi Arabia; 2 Department of Sociology and Social Work, Faculty of Arts and Humanities, King Abdulaziz University, Jeddah 21589, Saudi Arabia; 3 Department of Psychology, Oakland University, Rochester, MI 48309, USA

**Keywords:** parental selection, preschool, semi-structured interviews, Saudi Arabia

## Abstract

A global initiative has emerged in recent years to highlight early childhood education and care as a foundation for later learning and development. The goal of this study was to investigate the choices that Saudi Arabian parents made for their children’s preschool education. In a qualitative, exploratory study, we investigated parental selection of preschool programming in Saudi Arabia. Guided by a phenomenological approach, we conducted semi-structured interviews with 12 Saudi Arabian women that had preschool-aged children. Although some research has examined the constraints and factors affecting parental selection of preschool programming, it remains unclear when these constraints and factors are considered by parents during the decision-making process, and how the decisions are made. The interviews revealed that parents consider not only educational reputation in the selection of preschool programming, but also instructional use of Arabic and English, religious foundation, teaching quality, classroom preparation, program scheduling, tuition costs, and physical security of the preschool environment. The results suggest that improving information collection is essential for empowering parents to make wise decisions, with the child’s happiness and academic growth having a significant impact on those decisions. The discussion addresses the importance of identifying the factors that affect parental selection of preschool programming, so that educational professionals may better meet the needs and expectations of parents in Saudi Arabia.

## 1. Introduction

### 1.1. Benefits of Early Childhood Education

The benefits of early childhood education have been summarized by Field [1] and Allen [2]. Both scholars emphasized the need for educators to use early intervention as an approach for safeguarding long-term benefits to society. Appropriate and effective early childhood education is increasingly important as today women more frequently enter the workforce and seek custodial care for their preschool-aged children [3].

The values and beliefs of parents affect their selection of a childcare environment [4]. Although the values of parents are not necessarily clearly articulated, they inform parents’ perceptions in terms of which type of childcare they will use, how they react to different childcare environments, and how satisfied they are with them [5].

### 1.2. Early Childhood Education in Saudi Arabia

Saudi Arabia has recently enacted several policies regarding non-compulsory early childhood education. The relevant goals are to introduce children to a school atmosphere at an early stage rather than leaving them to experience only the conditions in the home environment [6]. Nevertheless, early childhood education enrollment in Saudi Arabia, which is estimated at 10–12%, is still low when compared with other countries, notably the United Kingdom (UK) and the United States (US) [6].

Although some previous research has explored parental knowledge of childcare practices, no previous research has investigated parental perceptions of preschool programming in the Saudi Arabian context, paying special attention to identifying the factors that parents consider important when making decisions about preschool programming for their children. Thus, the current qualitative, exploratory study investigated Saudi Arabian parental views on preschool programming for their children, with the goal of identifying the factors that affect parental decisions about where to enroll their children.

### 1.3. Related Research in the United Kingdom and the United States

In the UK and the US, the respective governments have renewed a focus on parental choice of preschool education for their children [2]. Instead of reducing funding for childcare, the relevant departments have left the decision-making process to market dynamics and parents, so that parents may identify the type of preschool service provisioning that most effectively facilitates the desired learning outcomes [7,8]. Although most research on preschool quality addresses quality from the standpoint of the service provider [9], less is known about how parents understand quality and how their perceptions about quality determine their selection of preschool programming.

The factors that affect the parental selection of preschool programming have been indirectly addressed in studies conducted in the UK as part of a times series data collection effort intended to review the effectiveness of government policy directives (1997–2010) [10,11]. Additionally, under the UK Childcare Act [12], local agencies are responsible for conducting Childcare Sufficiency Assessments (CSAs) [13], which are informed by local studies of parents. CSAs are designed to capture supply and demand to allow local authorities to augment access to childcare provisions while supporting providers to remain financially viable. The assessments revealed that there are inefficiencies in provision, with parents highlighting a lack of information and citing cost as a barrier to enrollment [14]. These concerns continue to appear whenever parents are surveyed [8,15].

The views and experiences of UK parents, with respect to formal and informal preschool programming, were assessed by Bryson et al. [11]. The findings documented how parents’ use of preschool had changed, with more parents opting to use formal preschool than in the previous decade [16]. The findings were used to advocate increasing the use of recognized preschool programming in line with prevailing policy directives. The majority of parents using formal preschool programming reported being satisfied with the provisioning, although it was reported that half of the formal providers could improve a number of aspects of service delivery. This corroborates findings in the literature on parental satisfaction in the US [17], with factors such as hours of availability, quality of facilities, and communication being heavily featured. According to the Bryson et al. study, the educational and social development of the child, coupled with well-trained educators, were key factors affecting parental selection of programs. Aspects such as program reputation and reliability also were cited as important in parental selection of programs, as well as the extent to which programs favored parents’ work schedules and had convenient locations [11].

According to the 2011 Daycare Trust survey [8], UK parents ranked the following factors as important in the selection of preschool programming: well-qualified, trained, and experienced teachers (74%); a warm and caring atmosphere (59%); a good OFSTED Report (44%); and cost (36%). The report concluded that enhancing the information-gathering process is central for enabling parents to make informed decisions, with the child’s happiness and educational development strongly affecting their selections. Moreover, according to Alexander [18], a successful child is a happy child. According to Cottle and Alexander [19], the values and goals held by practitioners for children are important determinants of educational quality. However, in the UK and the US [15,20,21], cost continues to play a major role in parental choices, over and above the role of quality. Furthermore, parents have been perceived to be uninformed as to which aspects determine their children’s success [22]. Although cost factors have been addressed to some extent with programs such as tax credits and early learning for two-year-olds, there remains a tension between the cost and adequacy of the programs and facilities [23]. The consumer model of preschool provisioning may enable parents to broaden their choices, with the expectation that service providers will continue to improve to meet the parents’ and children’s needs. The practical and political implications of ensuring sufficient high quality preschool education highlights the need to investigate and identify the factors that affect parental selection of preschool programming.

### 1.4. Aims and Objectives

The current research was a qualitative, exploratory study intended to investigate how Saudi Arabian parents arrive at decisions regarding the choice of preschool programming for their children. Identifying the factors that affect the parental selection of preschool programming may inform educational professionals and policy developers so that they can better meet the needs and expectations of parents in Saudi Arabia.

## 2. Materials and Methods

### 2.1. Study Design

This study used a constructivist phenomenological paradigm, which is frequently employed for naturalistic studies that describe the exploration, reconstruction, or comprehension of a phenomenon from the perspective of the participant [24]; most qualitative studies use some variant of this paradigm [25]. The use of a constructivist phenomenological paradigm recognizes that participants have various and differing worldviews and that, in this way, there may be several perceived realities. As a result, in line with the main research question, this study investigated parental perspectives on preschool programming.

### 2.2. Study Approach

The study used a qualitative approach to allow for an in-depth and more detailed understanding of the factors that affect parental selection of preschool programming. A qualitative approach allows the researcher to secure a rich and often more comprehensive understanding of a phenomenon [26]. In the current study, the focus was on identifying information available to and considered by parents during the selection of preschool programs, as well as the factors that contribute to their selection of preschool programs. In addition, the use of an inductive approach facilitated the opportunity to obtain more detailed and in-depth responses from participants [27].

### 2.3. Study Sample

The study sample comprised a purposive, non-random sample of Saudi women, specifically mothers with a child enrolled in a preschool program. This sampling method was used to allow for the collection of comprehensive and in-depth information and was chosen due to the ease of access to the study community. Interviews were conducted with 12 women, whose ages ranged from 25 to 45 years, whose educational qualifications ranged from a bachelor’s degree (8 women) to a masters degree (4 women), and the majority of whom were employed outside the home (11 women). The interview lasted between 45 min and 1 h. The participants were interviewed in locations that were comfortable for them so that they felt free to express their opinions and experiences concerning their children and the preschool program. We did not secure information about the socioeconomic status or class of the participants. Time constraints and the other research and teaching commitments of the researchers necessitated that the data collection would be concluded following completed interviews with 12 participants. Interviews were conducted from August 2020 to December 2020. Table 1 presents the available descriptive characteristics for the sample. The names of the participants are pseudonyms to protect the identities of the participants.

### 2.4. Study Tool

This study adopted a semi-structured interview method for several reasons. First, this method allows the researcher to investigate specific aspects of a phenomenon, allowing for the opportunity to delve more deeply into the research topic. Second, semi-structured interviews allow participants the relative freedom and time to express, in their own words, their experiences, ideas, and points of view without restrictions. Third, semi-structured interviews are flexible, enabling the researcher to direct or clarify questions to the participants in addition to adding or removing questions as needed [28]. A semi-structured interview guide was created in both English and Arabic; it consisted of open-ended questions and was pilot tested on two respondents; minor modifications were made afterwards to improve the clarity of the questions. The interviews were broadly guided by two questions: What information is available for parents to use in selecting preschool programs? What are the main factors that determined the selection of preschool programs?

### 2.5. Ethical Considerations

The research proposal for this study was reviewed and approved by the King Abdulaziz University Ethical Review Committee (protocol code: IFPHI-379-324-2020; date of approval: 20 October 2020). Participants confirmed that they were willingly participating and signed a statement of informed consent, available in English and Arabic, prior to the interviews. There was no coercion to participate, and the participants were informed of their right to withdraw from the study at any point and for any reason without penalty. Anonymity and confidentiality were ensured through the use of pseudonyms and codes in the dissemination of results.

### 2.6. Accuracy and Veracity of the Data

It is obviously important to ensure the accuracy of the interview data [29]. Therefore, all interviews were recorded using an audio recorder after obtaining permission from the participants. This recording allowed the interviewers to return with the participant to the audio-recorded interviews to verify the meaning and accuracy of responses.

### 2.7. Data Analysis

A qualitative analysis relies on an in-depth and rich interpretation of the content [30]. Qualitative analyses focus on and are concerned with interpretation, and there are many steps to achieve this: arranging data, engaging with data, developing ideas or elements, coding data, providing explanations through analysis, and finally collecting the results in a report [31]. Coding is an essential step of a qualitative analysis. Coding identifies broadly defined concepts or elements that are relevant to the phenomena of interest [32]. The intention of coding is to identify ideas and topics, prepare data, and organize data to allow for a detailed and in-depth analysis.

All transcribed audio interviews were copied into a Microsoft Word document, then the data were manually reviewed to ensure that information was not lost. The data were then read several times to outline keywords and identify repeated statements. Finally, the data were organized and arranged in a table, facilitating the presentation of the results in a narrative style.

## 3. Results

To identify the factors that affect the parental selection of preschool programming, we generated open-ended questions. These included questions about what information is available to parents that helped them select a preschool program, how a program is identified, and we posed a direct question about the factors that the parents considered when selecting a program.

As noted (and see Table 1), the ages of the participants were 25–45 years old, all were mothers of preschool-aged children, and most were employed outside of the home. These participants were purposefully selected because of the role and importance that the mother has in raising and guiding children, especially in the early stages of childhood. It should be noted that the cultural divisions between and the different tasks expected of the sexes in Saudi society give women more responsibility for the caring and raising of their children, in addition to working outside the home.

Through the responses of participants to the study questions, several factors were identified as important in the parental selection of preschool programming, including the information available for considering and selecting a preschool program. We address these factors in the next section.

### 3.1. Information Available to Parents

When the participants were asked about the information available to select a preschool program, they could be divided into two groups based on their responses. The majority relied on family and friends to obtain information, and the remainder relied on researching the programs.

The majority of the participants reported that they relied on the available information and experiences of other parents with preschool-aged children, especially the experiences of female relatives and close friends. One of the participants, Bushra, recalled the source of her information about the program that her daughter joined: “Through my sister, she is a teacher in the elementary school, and she provided me with information about the school’s interest in students and the quality of education they have in the preschool program”. Doaa also said that she followed her sister’s suggestions, noting, “I took my sister’s experience with her children in the preschool program”, meaning that her choice depended on the information provided by her sister. The experiences of female relatives who are also parents was an important factor that affected parental selection of a preschool program. These sources are considered to be especially reliable by the participants as they are perceived to be very likely to share all negative and positive experiences related to the program, giving parents a comprehensive assessment of the preschool environment.

Some of the other participants relied on self-guided research and obtaining information on their own initiative from the program administration. Bdoor stated that she got to know the preschool program she selected in this way, saying, “Close to my workplace, I went to the headquarters of the program and went to the administration. I asked them about the type of program, the fees, the educational and administrative staff, and some of the policies related to the program”. Thus, some mothers do not rely solely on relatives or friends for information about preschool programs and seek to acquaint themselves with a program’s management and educational staff.

In a different context, Sawsan sought information on preschool programs in a different way, saying, “We contacted the Ministry of Education to provide a list that includes the names of preschool programs that are accredited and trusted by them”. The focus on preschool programs accredited by the Ministry of Education is notable; preschool education programs are subject to permanent supervision to ensure their quality and to ensure that they perform their role in accordance with educational policies, rules, and regulations. In other words, this participant sought to ensure a healthy and sound environment for her child, one that was in line with the regulations, standards, and values of Saudi society.

### 3.2. Determinants of Program Selection

When directly questioned about the factors that play an important role in the selection of a preschool program, most participants identified a religious foundation, Arabic language use, English language instruction, teacher quality, student preparation in the classroom, hours of operation and instruction, program work, tuition and fees, and safe and child-friendly environment. We discuss these factors next.

#### 3.2.1. Religious and Linguistic Establishment

The majority of participants identified that teachings from and about the Qur’an were an important determinant of selecting a preschool program for their children. For example, Nada emphasized the use of Arabic language and teaching of the Qur’an as factors that were important in determining the preschool program for her child: “The program relies on focusing on the Holy Qur’an, so that they had specialized teachers, one of them for the Qur’an and the other for the Arabic subject, and as for me, the correct foundation for my child is the Arabic language”. Aziza also emphasized religious values in determining her child’s preschool program. She recalled being proud of her son, “because he repeats Islamic remembrances, whether when leaving the house, eating, etc”.

A few participants noted that the teaching of and use of the English language was a key determinant in selecting a preschool program. Bdoor said, “The most important thing for me is that the preschool program is international and teaches the British curriculum because it is strong in both spoken and written English”. In the same context, Sawsan considered the primary determinant for choosing a preschool program as, “the English language and also for teachers to speak English as their native language”. This focus on English language use as a determinant of preschool program selection by a minority of the participants in this study is surprising. However, the English language has gained increased importance in Saudi society, and thus mastering it is now perceived by many parents as an important life skill. This applies to children, especially in early education. Thus, in hindsight, some parents seek this feature of preschool programming so that their children can keep pace with the rest of the world, given that English is the primary language in most developed countries. Mastering the language will contribute to consolidating and enhancing their ability to acquire related language skills, such as reading, writing, listening, and speaking.

#### 3.2.2. Educational Qualifications of Teachers

Many of the participants emphasized that a strong factor in their selection of preschool programming was that the program had staff with high-quality educational training and experience. Bushra inquired about the qualifications and specialization of the teachers who would instruct her daughter through the program agent before registering. She said, “I learned that the teachers have experiences and special education in kindergarten and they have specializations in general psychology and also in developmental psychology, which increased my desire to register with them”. In the same context, Mashael said that she visited a program twice, adding, “I discovered many teachers’ attitudes related to the children and how they dealt with children, that they have experience and qualifications, so my heart was reassured by my daughter’s registration”. We highlighted the importance of the teachers’ specialization and experience because the parents perceive teachers to provide a vital role in the educational process. We also noted that the importance of experience does not diminish the importance of their mastery of the scientific material. In other words, experience refers to the mastery of both the educational material and methods of teaching. The teacher must understand the information and be able to effectively communicate it.

#### 3.2.3. Number of Students in a Class

Many participants stressed the importance of having only a small number of students in the classroom, as they felt that this is important for the interaction of the children with the teacher, as well as the teacher’s ability to sufficiently attend to all of the children in the class. Nada reported that one of the main factors that affected her selection of a preschool program for her daughter was the small number of students in the class. She added, “One of the daughters of my close friend is in one of the preschool programs, and the class contains more than 25 students. Her child was suffering from a hearing deficiency and the matter was exacerbated by the students, so that the teacher always complained about the child’s neglect and not hearing the directions, which led to the neglect of this problem of the child and delayed solution to the problem that the child suffers from”. In the same context, Bushra stated her expectation “that the number of students in the classroom does not exceed 12 students”. Furthermore, she said, “Every child has potential and abilities, and the teacher must focus on all children and give each child the opportunity to develop his or her talents”.

#### 3.2.4. Working Hours

Many of the mothers participating in the interview discussed the importance of the working hours of the preschool program as a factor in their selection of a program. This is because almost all participants are employed outside of the home, thus it is important for them to find a program available during the morning and noon periods. Aziza said, “The program availability is suitable for me as a working mother, as they operate from 7 a.m. to 9 p.m. If I am late in my shift, I am reassured about my child being able to stay with them”. Working mothers want to leave their children in a school that cares for them so that they are reassured and do not have concerns if, for example, they are late in picking up their children.

#### 3.2.5. Preschool Program Site

Many of the interviewees mentioned another determinant of selecting the program, namely the location and proximity to the parents’ workplace for easy access. Bdoor said, “The proximity of my child’s preschool program to my workplace is a factor that was important in selecting it”. Many mothers emphasized the importance of the school’s location in relation to their workplace, which offers both convenience and the ability to save time and money from reduced travel.

During the interviews, a few participants highlighted another component that was important in selecting a preschool program, namely that it was affiliated with and located in their workplace. Mona expressed how the program’s location at the workplace “allows me to check on my daughter, as my daughter suffers from diabetes, so I can be close to her and can follow her continuously in taking medicine and the quality of food, and also provide support and share with her during her school day”. We note that some mothers have an especially strong attachment to their children, especially if the child suffers from a specific disorder or disease. Thus, for this specific participant, having her daughter in a program at her workplace makes her feel close to her child, allowing her to check on her daughter during working hours and giving her feelings of reassurance and comfort.

#### 3.2.6. Tuition and Fees

For a group of participants, tuition costs played an important role in determining the preschool program. Doaa, for example noted, “The cost of the program is very reasonable, which means approximately 12,000 Saudi riyals per year for the child”. In contrast, Sawsan stated that the high costs of a preschool program helped her select that program. She stated, “The high costs indicate the high social and economic level of the students attending the school and they are the ones who will engage with my child and influence and be affected by them”.

#### 3.2.7. Safety and Appropriateness of Environment

Most of the interviewees noted the importance of a safe and appropriate environment for their children as a determinant of selecting a preschool program. They specifically referred to the building and the availability of emergency exits, the presence of a large roofed yard, protection from the sun, classroom capacity, and first aid facilities. Sawsan recalled that, in part, it was important for her to select a preschool program based on “the modernity of the building and its continuous maintenance, as well as the presence of entrances and exits for emergencies in the event of accidents or fire, the availability of fire extinguishers, and the presence of a private room equipped with first aid”. Sawsan also expressed, “My child spends about 8 h a day inside the building, and all of that makes me comfortable with my child there”. In the same context, the role of an appropriate environment for the child is important in selecting a preschool program for many interviewees. Bushra stated, “I found large playgrounds; their classrooms are large and equipped with modern means, … and when I took a tour of the school, I found that, so I was excited to register my child”. Meanwhile, Doaa said, “The squares are large and all of them are roofed to protect children from the sun”. Many participants noted that the school is the second home in which the student spends most of their hours of activity; thus, a healthy and safe environment is an important criterion for selecting a preschool.

## 4. Discussion

The results of this study revealed that the majority of respondents relied on family and friends for information about preschool programs. Furthermore, partly basing their selection of a preschool program on the previous experiences of relatives and trusted parents increased the confidence and security of the parents in their selection. A minority of respondents relied on their own research and inquiries about the programs.

The results of this study suggest that Saudi mothers focus on academics, religious instruction, teaching quality, the school’s characteristics, environment and facilities, convenience, and safety. However, several studies have shown that academic priorities are often paramount [33]. The current study showed that among the top priorities for mothers in Saudi Arabia (in terms of a preschool program) are the religious foundation of the preschool, including the teaching of Islamic principles, and the teaching of the English language. The latter factor is in line with a general trend in Saudi Arabia, with the Saudi Ministry of Education recently approving the teaching of English from the first grade of primary school [34].

Other results from the current study indicate that the parental choice of preschool program is affected not only by factors related to the location and cost, but also by those associated with the quality of teaching, as is reflected in the literature that has addressed Western preschool selection [35,36]. Meanwhile, parental interest in geographical convenience supports the idea that parents consider the accessibility and proximity of the preschool to their place of employment.

Many participants in the study expressed their desire that the preschool programs appoint educationally qualified people to teach rather than relying on high school graduates. This is consistent with the results of research with Western parents, which documents that parents’ decision-making about a school is affected by the quality of the academic staff. Western and Saudi parents alike prefer professional academic teaching staff who are highly skilled, on the presumption that such staff are best able to educate their children [37,38,39].

Parents in this study stressed the importance of having a small number of students in a class, so that their child receives sufficient and directed attention as well as sufficient care and time. To a degree, preparing the students in a classroom depends on reducing the size of larger classes—as a form of educational reform—which increases the number of individual interactions between the student and the teacher. The aim is to improve student learning and achieve order and discipline in the classroom while providing an atmosphere that allows for dialogue and discussion that respects the opinions of others. This facilitates equality in the classroom, so that each child receives appropriate attention, care, and time. This supports the findings of previous studies of Western parents on the teacher–student relationship (e.g., [40,41]). Small class sizes facilitate more effective interactions between the teachers and the students. In the context of Saudi Arabia, this is also perceived to be conducive to enhanced student performance. Al-Ansari [42] showed how the class size affects the relationship between Saudi students and the teacher by allowing the latter to employ novel teaching methods to encourage students to develop creative thinking skills. Small class sizes also enable teachers to become more familiar with their students and to focus on improving each student’s work ethic and positive thinking skills while solving problems.

The program’s operating hours and suitability with respect to the parents’ employment commitments is important, as is the location of the program and its proximity to the parents’ workplace. Proximity to the workplace reduces transportation time and costs. Moreover, a report by the International Labor Organization [43] proposed in-house childcare centers sponsored by the employer as a feasible solution to the childcare challenges of employees.

The importance of a safe and appropriate environment for the child resides in the fact that it serves as a second home for them, and it is the place where they spend much of their waking hours. A healthy and safe environment is important, meaning that emergency exits and first aid must be available, while the presence of covered yards for protection from the sun is an additional criterion that was identified by Saudi parents for the selection of a preschool program. Badri and Mohaidat [44] contended that the primary concern of parents was that their child was in a safe environment. Thus, the security of the institution instils confidence in both the parents and their children. A safe school environment is also important for parents as it believed to affect their children’s performance. According to Rehman et al. [45], the environment is among the most important factors in a quality education. However, Peterson et al. [46] found that school facilities only have a minor influence on student performance. Meanwhile, Bruce [47] found that a safe and appropriate school environment empowers students to demonstrate their abilities to the fullest, highlighting the importance of a school’s physical environment.

### Limitations and Future Research

The current study was unable to explore how parents from various socioeconomic backgrounds select preschool programs. By the researchers’ subjective estimation, most of the interviewees in the current study were middle class. Hence, additional studies on this topic could produce a more comprehensive understanding of this area. Furthermore, we only interviewed a small sample of 12 women. Future research may assess a larger sample. Finally, we encourage future research to investigate factors that affect fathers’ preferences for preschool programming. We attempted without success to recruit men as participants. Although we anticipate many similarities between the factors identified to be important by mothers and fathers, it is possible that men and women may weigh some factors differently; for example, fathers may weigh tuition and fees more heavily than mothers.

## Figures and Tables

**Table 1:** Characteristics of the participants.

Participant	Age	Marital Status	No. Children in Program	Education	Employment
Doaa	35–40	Married	1	Masters	Employed
Mashael	35–40	Married	1	Bachelor’s	Employed
Randa	25–30	Married	1	Masters	Employed
Nada	35–40	Married	2	Bachelor’s	Employed
Mona	30–35	Married	1	Bachelor’s	Employed
Ghadeer	30–35	Married	1	Bachelor’s	Student
Sawsan	40–45	Married	1	Bachelor’s	Employed
Bdoor	35–40	Married	1	Bachelor’s	Employed
Aziza	35–40	Married	1	Bachelor’s	Employed
Noor	35–40	Married	2	Bachelor’s	Employed
Bushra	30–35	Married	1	Masters	Employed
Afrah	30–35	Married	1	Masters	Employed

## Data Availability

Due to the nature of this research, participants did not agree for their identified data to be shared publicly.
